# MAP Kinases Pathways in Gastric Cancer

**DOI:** 10.3390/ijms21082893

**Published:** 2020-04-21

**Authors:** Lucia Magnelli, Nicola Schiavone, Fabio Staderini, Alessio Biagioni, Laura Papucci

**Affiliations:** 1 Department of Experimental and Clinical Biomedical Sciences, University of Florence, Viale G.B. Morgagni 50, 50134 Firenze, Italy; nicola.schiavone@unifi.it (N.S.); alessio.biagioni@unifi.it (A.B.); laura.papucci@unifi.it (L.P.); 2 Department of Experimental and Clinical Medicine, University of Florence, Largo Brambilla 3, 50134 Firenze, Italy; fabio.staderini@unifi.it

**Keywords:** MAPK, gastric cancer, metastasis, miRNA, lncRNA, epigenome

## Abstract

Gastric cancer (GC) is turning out today to be one of the most important welfare issues for both Asian and European countries. Indeed, while the vast majority of the disease burden is located in China and in Pacific and East Asia, GC in European countries still account for about 100,000 deaths per year. With this review article, we aim to focus the attention on one of the most complex cellular pathways involved in GC proliferation, invasion, migration, and metastasis: the MAP kinases. Such large kinases family is to date constantly studied, since their discovery more than 30 years ago, due to the important role that it plays in the regulation of physiological and pathological processes. Interactions with other cellular proteins as well as miRNAs and lncRNAs may modulate their expression influencing the cellular biological features. Here, we summarize the most important and recent studies involving MAPK in GC. At the same time, we need to underly that, differently from cancers arising from other tissues, where MAPK pathways seems to be a gold target for anticancer therapies, GC seems to be unique in any aspect. Our aim is to review the current knowledge in MAPK pathways alterations leading to GC, including *H. pylori* MAPK-triggering to derail from gastric normal epithelium to GC and to encourage researches involved in MAPK signal transduction, that seems to definitely sustain GC development.

## 1. Introduction

Every cell in our body is capable to respond to external stimuli such as growth factors, inflammation, cytokines, microenvironmental changes, and to interact with other cells, owing to a complex molecular mechanism based on the action of the mitogen-activated protein kinases (MAPKs). MAPKs are a large family of serine/threonine kinases that, upon the reception of a stimuli, trigger a cascade of phosphorylation leading to a precise and specific cellular response. Commonly the canonical pathway starts with the MAPKKKs, among which Raf isoforms are the better known and the most described in the scientific literature [1], that phosphorylate and activate the MAPKKs. To this family belongs the MAPK/ERK kinase (MEK) which plays a fundamental role in the activation of ERK1/2, which are the first mammalian MAPK genes to be identified in the late 1990s [2]. Even though ERK1/2 are the most studied members of the MAPKs family, many other are described to be involved in cancer development such as ERK3, ERK4, ERK5, and ERK7/8 [3,4]. MAPK pathway can be dysregulated in many ways in gastric cancer (GC), their components being in turn affected by trans-regulating components such as drugs, ligands, or endogenous proteins indirectly related to the pathway, or directly, because of mutations occurring in the components of the pathway itself. Noteworthy, important MAPKKK such as RAF and its binding factor RAS are often mutated in gastrointestinal tumors [5,6]. Moreover, the picture is further complicated since non-coding RNA popped up. In particular, micro RNAs (miRNAs) and long non coding RNAs (lncRNAs) have been discovered to intervene in regulating the MAPK pathway at different levels [7,8]. The regulation of such a complex mechanism is fundamental in tumorigenesis and metastasis. Indeed, many types of cancers present a dysregulated MAPKs system, altering many cellular properties such as metabolism, cell proliferation, apoptosis, and migration [9].

## 2. The Gastric Cancer Biological Complexity

**Figure 1:**
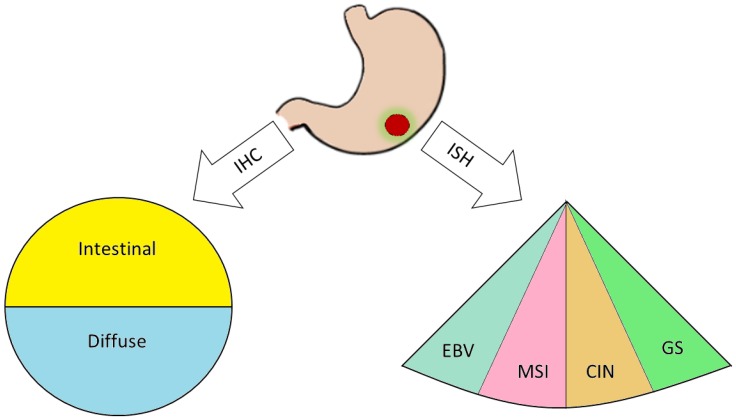
After tumor resection via surgery, the biopsy is processed through immunohistochemical (IHC) protocols, leading to a classical Lauren histopathological classification and/or exploiting in situ hybridization (ISH) techniques to evaluate EBV positivity (EBV), microsatellites instability (MSI), chromosomal instability (CIN), and genomical stability (GS).

GC which is actually the fifth most frequently diagnosed cancer and the third leading cause of cancer death according to GLOBOCAN 2018 [10], demonstrated in the past two decades a high rate of metastasis particularly localized in the liver (48% of metastatic GC patients), peritoneum (32%), lung (15%), and bone (12%) [11]. Metastasis formation, which is a very complex biological mechanism with hundreds of proteins involved, is reported to be regulated not only by focal adhesion on the cell membrane but also by matrix metalloproteinases (MMPs), which in turn are able to degrade the extracellular matrix allowing the intravasation of cancer cells into the endothelial or lymphatic vessels [12]. Currently, one of the few targeted therapies available for advanced GC is the use of Trastuzumab [13] combined with fluoropyrimidine plus cisplatin, when the amplification of the HER2 gene is revealed by liquid or solid biopsy. Indeed, HER2 is another important oncogene that is able to promote invasion and metastasis by stimulating selectively the RAF/ERK pathway and thus activating oncogene such as c-Fos, elk1, and c-Myc [14]. The majority of GC are adenocarcinomas which can be classified by different criteria. Among these, the Lauren histopathology system, which is still widely used, distinguishes two principal subtypes: namely, the intestinal and diffuse subtypes. Tumors that do not fit these types are considered mixed and indeterminate subtypes [15]. This classification is also epidemiologically relevant. Indeed, intestinal GC, which is more common in elderly patients, mainly males, is more often associated with intestinal metaplasia and *Helicobacter pylori* infection, and metastasize mainly to the liver through blood flood. Diffuse GC most commonly occurs in young patients, mainly females, presents a hereditary component and metastasize through peritoneal surfaces. Moreover, the diagnosis of diffuse GC is a pejorative prognostic factor, since it behaves more aggressively than intestinal GC. The extraordinary molecular heterogeneity of GC that has been highlighted by studies on somatic copy number alterations, gene mutations, epigenetic, and transcriptional changes etc., [16], led to a new classification. In 2014, the milestone study carried out by the Cancer Genome Atlas (TCGA) research network created a molecular-based stratification method that classifies GCs in four groups: 1) Epstein-Barr virus-positive GCs with high DNA hypermethylation, frequent JAK2 and PD-L1 amplification and PIK3CA mutations; 2) microsatellite unstable GCs with DNA hypermethylation and MLH1 silencing; 3) genomically stable GCs with frequent CDH1 and RHOA mutations and correlated with diffuse morphology; 4) chromosomally unstable GCs with high TP53 mutations, tyrosine kinase receptors amplification and intestinal morphology. To better understand the dichotomy of the GC classification, we reported in Figure 1 the comparison between the classical Lauren histopathology system and the new molecular one. Consistent with elevated genetic instability, mutations found in GCs are extremely divergent and have no characteristic driver gene mutations. Moreover, it was reported that genes involved in the Ras/ERK signaling pathway, such as *FGFR2*, *KRAS*, *EGFR*, *ERBB2*, and *MET*, are amplified in a mutually exclusive manner in approximately 40% of GC cases [17]. In spite of the increasing knowledge of molecular biology of GC, surgical resection of cancer and extended lymph node dissection remain the only effective curative therapy for GC, as in 70–90% of cases, owing to chemoresistance, chemotherapy is unable to inhibit the tumor growth, invasiveness, and metastasis spread [18]. Here we focus on the most recent advancements in the dysregulation of the MAPK pathway in GC.

## 3. The ERK/MAPK Pathway in Gastric Cancer

**Figure 2:**
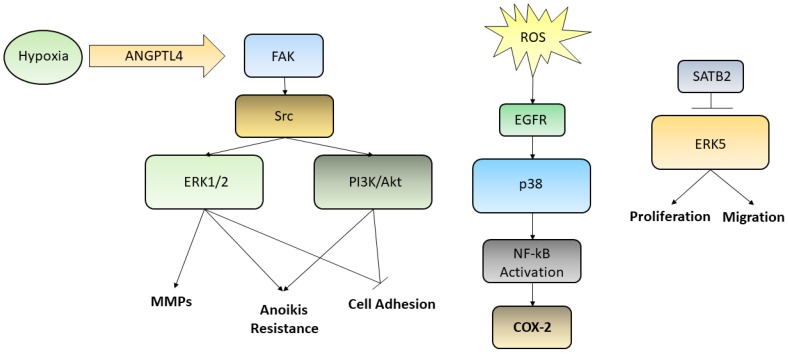
Although several kinases pathways are deregulated in GC, we reported here three ways by which ERK1/2, PI3K/Akt, p38, and ERK5 are activated. Hypoxic condition may trigger, through the ANGPTL4/FAK/Src/ERK1/2–PI3K/Akt axis, events aimed to increase GC cells metastatic potential, such as induction of anoikis resistance, MMPs secretion, loosening of cell adhesion; ROS, influencing EGFR and p38, are able to activate NF-kB translocation into the nucleus leading to the transcription of COX-2 gene; SATB2, which is commonly downregulated in poor prognosis GC patients, being able to inhibit ERK5, is capable to decrease cell proliferation and migration, acting as a tumor suppressor transcription factor.

In recent years, ERK-MAPK relationships have been abundantly studied, since mutations and/or alterations of activation pathways has been associated with neoplastic phenotypes of a large number of human tumor cells [19]. Various studies have demonstrated that the ERK/MAPK pathways are involved in the regulation of cell motility both in GC and in normal epithelia. Indeed, ERK regulates the activity of MMPs in GC, thus influencing cell migration and invasiveness [20]. Moreover, the secreted protein angiopoietin-like-4 (*ANGPTL4*), which is induced by hypoxia, exerts various effects on the neoplastic progression in scirrhous gastric carcinoma. GC cells may acquire resistance to anoikis through the activation of *ANGPTL4*-mediated FAK/Src/PI3K-Akt/ERK signaling, inducing the development of peritoneal metastases [21]. There are various studies that have correlated the alteration of the ERK/MEK pathway with the processes of invasion and metastasis; such processes involve motility and cell adhesion and epidermal growth factor receptor (EGFR)-induced disassembly of focal adhesions which is regulated by activating the ERK/MAPK pathways [22]. The p38 pathway turns out to be deregulated in many cancers as well. p38 signaling is involved in the regulation of the epithelial-to-mesenchymal transition (EMT) and in the production of reactive oxygen species (ROS) triggered by EMT which are significantly generated in GCs. One of the targets activated by ROS is EGFR whose overexpression has been linked to lymph node diffusion and consequently to a worse prognosis in GC. These studies have demonstrated the involvement of the EGFR/Ras/MAPK signaling pathway in the activation of NF-κB, in the induction of the cyclooxygenase-2 (COX-2) and in the proliferation of GC cells. NF-κB stimulates the transcription of COX-2, a regulator of cell proliferation. Upregulation of COX-2 facilitates the development of cancer and reduces apoptosis. High levels of COX-2 protein and mRNA have been detected in tissues of patients with GC. It is evident how the EGFR/Ras/MAPK signaling pathway is involved in the activation of NF-κB, through COX-2 induction, and in the stimulation of GC cells proliferation. Han et al. found that lycopene inhibits cell proliferation and induces cell apoptosis by reducing the levels of ROS, and thereby inhibiting ROS-activated EGFR/Ras/ERK and p38 MAPK signaling pathways and suppressing NF-κB p50/p50-mediated COX-2 gene expression in the Caucasian GC cell lines AGS [23]. On the other hand, a large number of studies on GC have shown that the MAPK pathway is associated with apoptosis and autophagy [24]. Liu et al. used ginsenoside Rg5, a rare saponin belonging to the protopanaxadiol ginsenoside family, used as a therapy against many types of cancers, in order to demonstrate its anticancer effect also in GC [25]. In this case Rg5 led to a ROS increase and activated the MAPK pathways, such as those involving p38 and JNK, that works as cancer suppressor, promoting apoptosis autophagy and blocking the cell cycle during the G2/M transition. Also, ERK activation contributed to ROS-associated Rg5-induced GC death. Finally, it was reported the special AT-rich sequence-binding protein 2 (SATB2) as a new tumor-suppressive gene playing an important role in many cancers, including GC. Downregulation of SATB2 in patients with GC was associated with shortened survival and, in the gastric cancer cell line MGC-803, the overexpression of SATB2 was able to repress ERK5 expression, while activation of ERK5 restored the SATB2-induced inhibition of proliferation and migration [26,27]. Even though we reported few examples of the relationships between metastasis formation and the abnormal activation of MAPKs in GC, describing the complex interconnection leading to anoikis resistance, cell adhesion loosening, MMPs secretion and activation, increased proliferation and migration, many efforts need to be still spent in order to elucidate every single pathway and thus uncover new possible targets for future therapies. The main pathways reported above were summarized in Figure 2.

## 4. Pharmaceutical Effects of MAPK Targeting in Gastric Cancer

Many studies reported a correlation between the efficacy of antiblastic drugs and the blockage of ERK1/2 activation. Indeed, the MAPK/ERK Kinase is deregulated in one-third of all human cancers. It is estimated that approximately 37% of patients with GC are potentially targetable by RTK/RAS-directed therapies [28]. Signaling pathway inhibitors are recognized to be effective and there are currently several clinical trials ongoing. For GC multiple classes of antibiotics were found to inhibit the ERK/MAPK pathway. A recent study repurposed 26 antimicrobials agents which were screened on AGS GC cell line for their potential to target the ERK/MAPK pathway [29]. Among these, the most effective was doxycycline, a widely used antibiotic, that acts by interrupting the tRNA binding to the 30S ribosomal subunit during translation. Doxycycline is involved in various processes such as induction of apoptosis, reversion of EMT by restoring the expression of E-Cadherin, blockade of the cycle in G1-S, stabilization of p53 and p21, regulation of cell adhesion and migration, modulation of the ROS/ASK1/JNK pathway. Pandian and colleagues have shown on AGS, MKN45, and KATO III GC cells that doxycycline inhibited ERK/MAP pathway, both at transcriptional and protein level. In such a way, doxycycline blocked proliferation, colony formation, the ability to form spheroids and also inhibited a factor, the HSP90, which triggered the upstream cascade. The pathways inhibited by doxycycline, such as the ERK/MAPK, ER, E2F, Myc, Wnt, Notch, SMAD2/3/4, and OCT4, are largely expressed in a specific subset of GCs, which justifies the repurposing of this drug Another well-known inhibitor is Metformin, which is a biguanide drug that is used in the treatment of type II diabetes and epidemiological studies have suggested that its use is associated with a decreased incidence of various types of cancer [30]. It has been reported to inhibit cell proliferation in human GC cell lines, including MKN45, MKN47, MKN-28, SGC-7901, and BGC-823 and reduce metastasis of human AGS cells, by inhibiting EMT in a glucose-independent manner [31]. A recent study shows how metformin inhibited viability and promoted apoptosis in AGS GC cells [32]. Metformin-induced apoptosis may be mediated via downregulation of ERK, JNK, and p38 phosphorylation. By decreasing insulinemia and glycemia, metformin can block the PI3K/MAPKs signaling pathway, which are implicated in cancer cell growth. Many natural products isolated from animals, plants, fungi, and bacteria are used for the treatment of various diseases, including cancer. Quercetin is a flavonoid identified in vegetables, fruits, and tea which inhibits p38, ERK, and JNK committing to death of AGS cells [33]. Li and Chen analyzed the effect of Quercetin on GC metastasis and the correlation of its effect with the uPA/uPAR system. The uPA/uPAR system is known to be critically important in GC metastasis development and high levels of uPA and uPAR may predict an adverse outcome in GC patients. In this study, Quercetin has demonstrated its antimetastatic effects on GC by blocking uPA/uPAR-dependent pathways. The suppression of the uPA/uPAR exercised by Quercetin may be related to NF-κb, PKC-δ, and ERK1/2 inhibition. Indeed, the latter is an important regulator, since its inhibition was associated with a reduced expression of uPA and uPAR. Fisetin is another flavonoid found in fruit and vegetables that has proven to exert an inhibitory effect on the proliferation of SGC7901 cells and induced apoptosis through suppression of ERK1/2 activation [34]. Another recent study has shown how Parameritannin A2, a product extracted from the stems of the plant *Urceola huaitingii*, has a synergistic effect with doxorubicin in GC cells. Doxorubicin is widely used as an antiblastic but induces chemoresistance and has cardiotoxic effects. Following treatment with doxorubicin, the PI3K/Akt, ERK1/2 and p38 signaling pathways were all activated but the synergistic treatment with Parameritannin A2 increased doxorubicin-induced mitochondria-dependent apoptosis [35]. Although many promising preclinical drugs are under development, currently only few clinical trials are ongoing on MAPKs targeting agents. Among the phase I concluded studied, we report the Triptolide, a compound extracted from the *Tripterygium wilfordii*, a Chinese medicinal plant, which effect is mediated by the suppression of oncoproteins, including DUSP1 [36], NF-κB, and heat shock proteins [37].

## 5. SPON2 and RAB13 as Two Novel MAPK Regulators in Gastric Cancer

SPON2, which is the secreting-type ECM Spondin 2 (SPON2, formerly Mindin, DIL-1), a member of the Mindin F-Spondin family, is highly expressed in many tumors but its molecular mechanisms and implication in cancer remains unclear although high levels of expression are correlated with a poor prognosis, migration, and proliferation [38]. SPON 2 levels are higher in metastatic GCs and SPON2 serum levels are higher in patients compared to healthy controls as well, and such levels increase as disease progresses. SPON2 promotes the EMT of malignant cells through the ERK1/2 pathway and the activation of signaling pathways such as the MAPK cascade, which contributes to relapse and metastasization of GC. p-ERK1/2 has been significantly reduced in SPON2 knockdown cells, although the levels of ERK1/2, p-MAPK8, MAPK14, MAPK8, and p-MAPK14 were not significantly different. Low SPON 2 levels inhibit EMT in GC deregulating ERK1/2 pathways and consequently affecting migration and invasion. Another non-canonical ERK1/2 regulator, RAB13, which is a member of the Rab GTPase family, is located in specific organelle membranes and vesicles, and cooperates to strictly regulate each step of the membrane traffic. Chen et al. examined the biological function of RAB13 in AGS GC cells and demonstrated that depletion of RAB13 attenuated the activation of Akt/ERK/mTOR signaling pathways through the inhibition of the expression level of EGFR in the plasma membrane [39]. All the three phosphorylated proteins are nodal in the signaling pathways supporting survival, growth, and proliferation in GC, and decreased levels of these proteins demonstrated that cells viability was hampered. This, at least partially, explains why RAB13 stimulates growth and proliferation via activating Akt, mTOR, and ERK pathways in GC cells.

## 6. *Helicobacter Pylori:* The Great Exploiter of MAPKs

**Figure 3:**
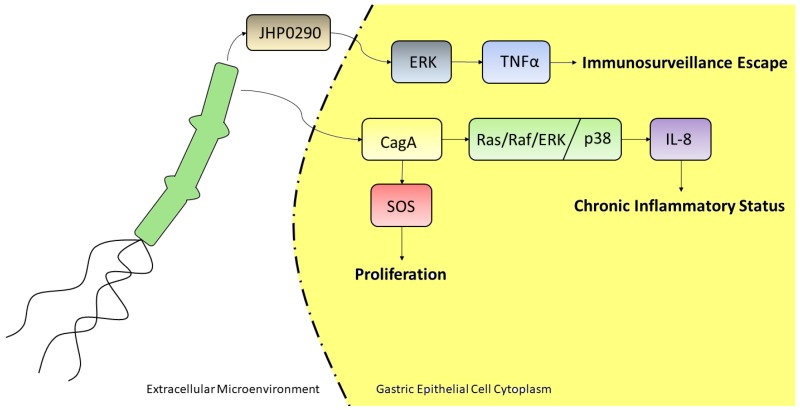
*H. pylori* may act as an oncogenic factor through several pathways and via many secreted proteins. In the above reported example, it is able to induce a chronic inflammatory status through the internalization of the CagA protein by gastric epithelial cells, which in turn triggers the upregulation of IL-8 via Ras/Raf/ERK or p38 pathway; such protein might also stimulate the Ras GTPase SOS, to induce cell proliferation. The secreted protein JHP0290 is able to induce TNFα transcription, mediated by ERK activation, leading to immunosurveillance escape.

GC is a multifactorial disease, whose risk factors are represented by complex interplays among pathogen, environmental and host-related factors [40]. Among pathogens, it is widely accepted that *H. pylori* infection plays a major etiological role in the development of GC, accounting up to 89% of the disease [41]. Since 1994, a three-cohort study led the WHO International Agency for Research on Cancer to classify *H. pylori* as a group 1 carcinogen [42]. Helicobacter pylori is a Gram-negative bacterium that is able to persistently colonize the human gastric mucosa, being fitted with enzymes and virulence factors allowing it to outlive the extreme conditions proper of the stomach [43]. First, on the eve of surviving, ingested *H. pylori* buffers unfavorable gastric lumen low pH through the secretion of the enzyme urease that converts urea into ammonia and bicarbonate. Subsequently, *H. pylori* uses its unipolar flagella to move across the thick mucus layer until it reaches the gastric surface. Owing to the triggering of immunosuppressive mechanisms, if not eradicated by antibiotic treatment, *H. pylori* colonization of gastric surface may become chronic, inducing an equally chronic inflammation state increasing cell turnover that, over time, can lead to the destruction of normal gastric glands (gastric atrophy) and their replacement with intestinal-type epithelium (intestinal metaplasia), being atrophic gastritis and intestinal metaplasia precancerous lesions that can progress to GC [44]. It is a matter of fact that host-*H. pylori* interactions are mainly mediated by virulence factors that trigger or alter MAPK signaling of host cells, to regulate proliferation of gastric epithelial cells [45], to escape immunosurveillance, and to generate a chronic inflammatory state. CagA (cytotoxin-associated bacterial protein A) is a 120-140 kDa unique protein, encoded by the cagA gene, which belongs to the cag pathogenicity island (cagPAI), together with other genes coding for proteins that assemble to form a type IV secretory system (T4SS) to export proteins outside the bacterium [46]. cagPAI is only found in highly virulent strains, with a rate between 90–95% in East Asian countries, and about 40% in Western countries [17]. CagA is considered a true oncoprotein, as its presence is associated with the appearance of precancerous lesions and a higher risk of developing GC [47]. Following the adhesion of the bacterium to the surface of the cell of gastric epithelium, the T4SS, acting like a syringe, delivers CagA inside the cytoplasm of the host cell. CagA, localized to the inner face of plasma membrane, induces hypermobility and morphological changes in the epithelial cell, that assumes the so-called “hummingbird phenotype,” which recalls that of cells that are treated with some growth factors. Indeed, when phosphorylated in apposite domains by host tyrosine kinases, like c-src and c-abl, CagA acts an impostor to recruit the SH2-containing domain SHP2 protein, a tyrosine phosphatase needed to fully activate the Ras/ERK MAPK pathway [48]. Tyr-phosphorylated-CagA lures SHP2, mimicking SHP2-recruitment by phospho-Tyr exposed by ligand-activated tyrosine kinase receptors (RTKs). This leads to an alteration of intracellular signaling, resulting in an increase of the proliferation rate of gastric epithelium cells, through a sustained activation of the Ras/ERK MAPK pathway [49]. Furthermore, Ras/ERK MAPK pathway can be directly activated by this viral factor in a phosphorylation-independent state. CagA can interact with an adaptor protein like GRB2, thus allowing recruitment of SOS, the RAS GTPase exchange factor, to the plasma membrane, thus stimulating cell proliferation and scattering [50]. The increase in the mutation rate because of hyperproliferation, together with a state of chronic inflammation, is consistent with the definition of tumorigenesis as a “wound that never heals.” Indeed, it is well-known *that H. pylori* infection induces the production of potent pro-inflammatory cytokines by different cell types [51]. Whole genome profiling of *H. pylori*-exposed gastric epithelial cells revealed that interleukin-8 (IL-8) is the most markedly up-regulated gene [52]. This cytokine, initially characterized as a potent recruiter and activator of neutrophils, later revealed to be a multifunctional factor, increasing angiogenesis, EMT, extracellular matrix remodeling, and macrophage activation in physiological conditions [53]. According to the notion that most gastric carcinomas are the end product of a chronic inflammatory state, the strong upregulation of IL-8 due to the exposure of *H. pylori*, supports a chronic inflammatory microenvironment that can eventually degenerate into a cancerous lesion [53]. Indeed, high levels of IL-8 in gastric mucosa indicate an increased risk of developing GC, and plasmatic level of IL-8 can be considered a diagnostic marker of this tumor [54]. *H. pylori* CagA induces epithelial gastric cells to produce high levels of IL-8 even at a low bacterial load infection [55]. Again, signal transduction to activate IL-8 gene expression occurs through the activation of ERK-MAPK pathway mediated by CagA. The promoter region of the IL-8 gene contains binding sites for transcription factors, such as NF-κb, and AP-1. CagA induces AP-1 by activating Ras/Raf/ERK MAPK pathway, both by P-CagA/SHP2- and CagA/Grb2-dependent pathways. At the same time, phosphorylated ERK activates NF-κb that translocate from cytosol to the nucleus to further activate IL-8 gene expression [56]. *H. pylori* can upregulate IL-8 levels also by the activation of p38 MAPK, but through a different mechanism; rather than inducing NF-κb translocation, p38 MAPK signaling induces the expression of p60 and p55 subunits that form the dimer of NF-κb [57]. Although *H. pylori* is highly immunogenic, the host fails to eradicate the infection in the majority of cases. This is partly due to the fact that this bacterium uses strategies to escape eradication through the modulation of the effector functions of the cells of the immune response, among which activated macrophages at the sub-mucosal space play a main role. JHP0290 and HP1286 are two poorly characterized proteins that are part of *H. pylori* secretoma. It has been demonstrated that these proteins are virulence factors able to bind to and induce macrophage apoptosis, through the ERK MAPK signal transduction pathway activation [58,59]. Furthermore, JHP0290 induces ERK MAPK-dependent production of TNF-alpha [58]. This is an acid-suppressive, pro-inflammatory cytokine. Together, these virulence factors contribute to create a favorable microenvironment, in which the bacterium can escape the action of macrophages, lower acidity, and support the host’s inflammatory response. About the involvement of *H. pylori* infection in gastric cancer progression, it was shown that this bacterium induces up-regulation of the expression of VEGF, the main pro-angiogenic factor, through the activation of p38 MAPK pathway in *H. pylori*-infected gastric cancer MKN45 cells [60]. All together such observations, summarized and represented in Figure 3, lead to define *H. pylori* as a risk for GC development, influencing deeply not only the proliferative status of the gastric epithelia but also inducing chronic inflammation and lowering the stomach pH.

## 7. MKK4 Involvement in Gastric Cancer Pathogenesis

MKK4 (also known as MEK4, SEK1, JNKK1, SKK1) is codified by the gene MAP2K4 located on chromosome 17p11.2 and is involved in several complicated signaling networks and might play different biological functions in different pathways, like cell proliferation and differentiation [61]. MKK4 has also been implicated in apoptosis and neoplastic transformation. The conflicting biological effects of MKK4 in human cancer might reflect the complexity of MAPK signal transduction. Genetic inactivation of the MKK4 gene on chromosome 17p has been reported in pancreatic, biliary, and breast carcinomas [62]. Furthermore, progressive loss of MKK4 expression has been reported in prostate, and breast cancers [63,64]. pMKK4 might function as a tumor suppressor in colorectal cancer. Downregulation of pMKK4 was associated with a more aggressive phenotype and with increases in local invasion and metastasis [65]. With regards to GC, MKK4 is mainly a tumor suppressor. Liang et al. show that curcumin inhibited cell proliferation and caused BGC-823 cells apoptosis via ROS-mediated ASK1-MKK4-JNK signaling pathways, but the activation of ASK1/MKK4/JNK cascade was effectively inhibited by the ROS scavenger N-acetylcysteine (NAC), indicating that the production of ROS in BGC-823 cells resulted in the signaling pathway activation [66]. A Chinese study correlated the impact of genetic variation on the general FAS signaling pathway with GC risk [67]. The genes examined in this pathway encode proteins involved in FAS receptor-ligand binding, initiator and effector caspases and structural proteins. They found genetic variations that contribute significantly to the overall risk of GC. Among these, MAP2K4 gene is significantly associated with GC. Polymorphisms in this gene have been correlated with risk association in a number of cancers in both Chinese and Caucasian populations, although several genetic discordances were highlighted between these two ethnicities.

## 8. Epigenetic Regulation via MAPK

Evidences suggest that ERK pathway is correlated with neoplastic progression, but the exact epigenetic regulation mechanism to date is not clear. It is important to point out that post-transcriptional modifications of the histones N-terminal can regulate chromatin organization and DNA utilization processes, including transcription. Studies in which histone modification has been correlated with tumorigenesis reported that deacetylases expression might be an independent prognostic marker in GC [68,69]. Eukaryotic DNA is surrounded by an octamer made up of four different types of histones, H2A, H2B, H3, and H4. H2A.X is the histone H2A family variant, and its phosphorylation at the site Tyr-142 can participates in multiple biological processes, including DNA repair. Numerous studies indicate that histone modifications are found in a high number of tumors and is considered very important in GC [69]. A recent study has shown that Ras-ERK signal pathway decreased H2A.X^Y142ph^ and promoted cell growth and metastasis, enhanced cell viability, cell colonies, and cell migration, explaining how Ras-ERK pathway could promote GC cell growth [70]. Another study highlighted that the activation of ERK1/2 signaling, induced by Ras mutation, might repress the phosphorylation of H1.4 at Ser27, blocking its protecting activity [71], elucidating the impact on the link between H1.4 Ras/ERK1/2-mediated phosphorylation and GC. Indeed, histone H1.4^S27ph^ has an anticancer activity, promoting the stability of the chromatin, and acts as a tumor suppressor in GC lines, controlling growth and migration. Being human DNA fine regulated by hundreds biological mechanisms, we do believe that worldwide research needs to proceed several steps forward in understanding how epigenetic regulation might influence the tumor progression through ERK/MAPKs pathways in GC.

## 9. miRNAs Regulating MAPK in Gastric Cancer

Micro RNAs (miRNAs), are short non-coding RNAs of approximately 20–22 bp reported to have a high impact on the regulation of several biological phenomena such as proliferation, migration, apoptosis, development, angiogenesis, immune response, and cell differentiation [72]. They are transcribed in the immature form of pri-miRNAs and processed into pre-miRNAs by a nuclear enzyme complex composed by DGCR8 and Drosha. Pre-miRNAs are then exported from the nucleus into the cytosol where they are further processed by the RNAse Dicer. Being the pre-miRNA composed by a -5p arm, a -3p arm, and a terminal loop, the cut made by Dicer commonly releases two mature miRNAs which are loaded on the ARGO protein complex, forming the so-called miRNA-induced silencing complex (miRISC), ready to inhibit the transcription, the translation or the lifetime of many target mRNAs [73]. Once loaded, ARGO recognizes the -5p arm or the -3p arm as “passenger” and after releasing the chosen miRNA, this one is degraded, while the other one called “guide” is used to identify the target mRNA. Having a pleiotropic effect on the regulation of several cellular pathways, miRNAs play a fundamental role in tumor biology and particularly it was reported that they modulate MAP kinases, such as Akt, ERK1/2, and JNK, affecting GC cells proliferation, survival, and metastasis [74]. Indeed, being genomic instability one of the main features of many types of advanced GC, it has been recently proposed a subclassification based on the miRNA profile [75]. Several dysregulated miRNAs are able to affect the cellular kinase pathways such as miR221/222, which can modulate PTEN and thus regulate PI3K activity, increasing cell proliferation and survival [76]. miR21 and miR214 exert the same effect regulating PTEN expression [77]. On the same PI3K-Akt pathway, miR375 targets PDK1, which in turn phosphorylates and thus activates Akt. Thus, overexpression of miR375 inhibits cell viability, leading GC cells to apoptosis [78]. miR375 was reported to target JAK2 as well, acting as a tumor-suppressor and inhibiting cell proliferation [79]. A more direct approach is exerted by miR143/145, interacting with Akt itself and so inhibiting proliferation and resistance to 5-fluorouracil [80]. On the other side, miR135b as well, is able to regulate the mammalian ste20-like kinase 1 (MST1) which in turn decreases the expression of p-p38MAPK, p-ERK1/2, P-glycoprotein, p38MAPK, ERK1/2, MDR1, MRP1, LRP, and Bcl-2, inhibiting GC cells resistance to cisplatin [81]. miR29 too, may regulate the ERK pathway interfering on the cell cycle [82]. When epigenetically silenced through hypermethylation, miR181c increased NOTCH2/4 and KRAS expression acting on the proliferation levels [83]. The study of miRNAs involvement in cancer is not only extremely interesting from a mere molecular point of view but is also an important topic for the development of future therapies. Indeed, owing to the more and more fine sequencing techniques, we are increasing the available knowledge on the mutations of many miRNAs, fueling a personalized medicine approach. In Table 1 are listed the miRNAs involved in MAPK regulation in GC.

## 10. lncRNAs Involvement in Gastric Cancer Modulation of MAPK Pathways

Beside microRNAs, another kind of non-coding RNAs are produced in the bulk of transcriptional output: the long non-coding RNAs (lncRNAs). The lncRNAs are usually RNAs longer than 200 base [84], but according to St Laurent (2013), it is difficult to standardize the ensemble of the non-coding RNAs because of “insufficient theoretical basis to classify and categorize the dark matter transcripts” [85]. Nevertheless, many lncRNAs are endowed with regulatory functions and their dysregulation is often associated to diseases [84]. A recent review [86] illustrates 16 different regulative functions performed by lncRNAs, from regulation of mRNA decay to sponging and neutralizing miRNAs. Among the diseases associated to lncRNA dysregulation, there are many degenerative diseases and cancers. In this panorama GC does not make an exception. Browsing the online lncRNA database at RNACentral [https://rnacentral.org/] 3474 entries, corresponding to lncRNAs associated to GC, can be retrieved. Very often, lncRNAs involved in GC act as a sponge for miRNAs. Some lncRNAs associated to GC are also responsible for GC overgrowth and survival mediated by the MAPK/ERK pathway. The very first report about the role of a lncRNA in GC is about antisense H19 [87]. H19 is a non-coding transcript able to sponge miR675 thus inhibiting the apoptotic process in GC [88]. However, miR675 in colon cancer has been found to promote migration and invasion via the MAPK signaling pathway [89], thus it is tempting to speculate that also in GC the MAPK pathway is involved. WT1-AS is another antisense expressed by the Wilm’s tumor gene [90,91]. The promoter of WT1-AS was found highly methylated and the antisense transcript downregulated in GC samples with respect to matched normal mucosa. Also, WT1-AS low expression level correlated with tumor size and clinicopathological stage. Ectopic expression of WT1-AS resulted in reduction of invasion and proportion of cell in G1 phase in in vitro experiments and in reduction of tumor growth and metastasis in in vivo experiments. Authors also demonstrated that WT1-AS overexpression resulted in ERK phosphorylation reduction and that a MAPK-based mechanism underlie the phenotypic effects. Another study in GC cell lines demonstrated a role for Yap1 protein, stably elevated in GC specimens, it concurs in promoting GC growth and metastasis controlling the expression of many lncRNAs and the phosphorylation status of ERK1/2 [92]. When upregulated Yap1 resulted in elevated ERK1/2 phosphorylation and increased expression of lncRNAs such as HOTAIR, H19, and MALAT1, respectively. This is the first report of an association between HOTAIR and MAPK pathway in GC but not the only report about involvement of HOTAIR in GC. Recently a role for HOTAIR as a miRNA sponge has been described both in GC, and liver fibrosis [93,94]. In particular HOTAIR acts as a decoy for miR29 and miR618, respectively. MALAT1 is another lncRNA acting as a sponge for miR124, and in retinoblastoma its overexpression results in ERK/MAPK and Wnt/β-catenin pathway activation [95]. SNHG6 is also a lncRNA responsible for JNK regulation in GC [96]. Like other lncRNAs listed above, there are reports suggesting that it acts as a sponge for miR-101-3P [97], a miRNA associated to the MAPK pathway [98]. lncRNAs is an emerging and very promising area of focus in GC, and as reported for the miRNAs’ world, recent sequencing profiling studies have identified several clues of association between such RNAs and GC. The deeper knowledge of their mutations and effects, will trigger a brand-new era for molecular biology and will start a new phase of drug development. In Table 2 are listed the lncRNAs involved in MAPK deregulation in GC.

## 11. Conclusions

MAPKs participate in complex biological systems accounting for an entire world of cellular kinases, that being activated by many stimuli, in turn influence many important pathways. Their understanding is of paramount importance, as in numerous cancers the kinases families are commonly dysregulated and, as reported above, they might be interesting targets for future therapies. GC in particular, showing several molecular discrepancies among different ethnicities, may be a very interesting model of study to further understand MAPK roles in cancer. Such discrepancies will need to be further deeply investigated, not only to select more specific therapeutic protocols, but also to better understand more thoroughly their molecular bases and effects. We do believe that, even if many findings are slowly uncovering the real impact of the MAPKs in GC progression and metastasization, much more efforts need to be spent in order to detect and evaluate all MAPKs regulators and effectors. We hope that this review article, as a little compendium of the most and recently studied topics about gastric cancer and MAPKs, will be a stimulus for new researches. Improving the worldwide scientific knowledge on the involvement of the dysregulated kinase pathways in GC will encourage also new studies on therapeutic drugs which might improve the survival of GC patients, exploiting such extremely important cellular kinases.

## Figures and Tables

**Table 1:** A comprehensive list of the most studied miRNAs regulating the MAPK pathways.

miRNA	Target	Effect	Reference
miR21	PTEN	Increases cell proliferation and survival	[77]
miR29	ERK	Inhibits cell proliferation	[82]
miR135b	MST1	Increases resistance to Cisplatin	[81]
miR143/145	Akt	Inhibit cell proliferation and 5-FU resistance	[80]
miR181c	KRAS	Inhibits cell proliferation	[83]
miR214/221/222	PTEN	Increases cell proliferation and survival	[76]
miR375	PDK1/JAK2	Inhibits cell proliferation	[78,79]

**Table 2:** An overall list of lncRNAs modulating the MAPK pathways.

lncRNA	Direct Function	Effect	Reference
HOTAIR	miR29 and miR618 sponging		[94]
WT1-AS	miR-330-5p sponging	Downregulated in GC. Inhibits proliferation and invasion	[90,99]
H19	miR675 sponging		[87,88]
AOC4P	Unknown	Upregulated in GC. Promotes proliferation, migration, invasion, survival	[100]
KCNKI5-ASI	miR21 sponging in gastric cancer	Upregulated in GC. Inhibits proliferation, promotes apoptosis	[101]
CASC2	Unknown	Downregulated in GC. Inhibits cell growth	[102]
MALAT1	miR124 sponging in retinoblastoma	Upregulated in GC. Promotes proliferation, migration, invasion, inhibits apoptosis	[95,103]
CARLo-5	Unknown	Upregulated in GC.	[104]
LINC01138	miR-1273e sponging in GC	Upregulated in GC. Promotes proliferation, invasion, migration, and inhibits apoptosis	[105]
lncNEAT1	miR-129-5p sponging in papillary thyroid carcinoma	Upregulated by solamargine in GC. Inhibits proliferation	[106,107]
DUSP5P1	Unknown	Upregulated in GC. Promotes cell proliferation, migration/invasion, and inhibits apoptosis	[108]
PICART1	Unknown	Downregulated in GC Inhibits proliferation and promote apoptosis	[109]
MAP3K1-2	Unknown	Upregulated in GC Promotes proliferation and invasion	[110]
Linc00483	miR-30a-3p sponging	Upregulated in GC Promotes cell proliferation, invasion and metastasis	[111]
SLC7A11-AS1	down-modulates sense sequence SLC7A11	Downregulated in GC Inhibits proliferation in GC	[112]

## Abbreviations

ANGPTL-4 CagA COX-2 EGFR EMT lncRNA MAPK	angiopoietin-like-4 Cytotoxin-associated bacterial protein A Cyclo-oxygenase-2 Epidermal growth factor receptor Epithelial-to-mesenchymal transition Long non-coding RNA Mitogen-activated protein kinases
MEK miRISC	MAPK/ERK kinase miRNA-induced silencing complex
miRNA MMPs ROS	Micro RNA Matrix metalloproteinases Reactive oxygen species

## References

[1] Yaeger R., Corcoran R.B. (2019). Targeting Alterations in the RAF–MEK Pathway. Cancer Discov..

[2] Bonni A. (1999). Cell Survival Promoted by the Ras-MAPK Signaling Pathway by Transcription-Dependent and -Independent Mechanisms. Science.

[3] Cargnello M., Roux P.P. (2011). Activation and Function of the MAPKs and Their Substrates, the MAPK-Activated Protein Kinases. Microbiol. Mol. Biol. Rev..

[4] Tusa I., Gagliardi S., Tubita A., Pandolfi S., Urso C., Borgognoni L., Wang J., Deng X., Gray N.S., Stecca B. (2018). ERK5 is activated by oncogenic BRAF and promotes melanoma growth. Oncogene.

[5] Mo S.P., Coulson J.M., Prior I.A. (2018). RAS variant signalling. Biochem. Soc. Trans..

[6] Yang Q., Huo S., Sui Y., Du Z., Zhao H., Liu Y., Li W., Wan X., Liu T., Zhang G. (2018). Mutation Status and Immunohistochemical Correlation of KRAS, NRAS, and BRAF in 260 Chinese Colorectal and Gastric Cancers. Front. Oncol..

[7] Tasharrofi B., Ghafouri-Fard S. (2018). Long Non-coding RNAs as Regulators of the Mitogen-activated Protein Kinase (MAPK) Pathway in Cancer. Klin. Onkol..

[8] Masliah-Planchon J., Garinet S., Pasmant E. (2015). RAS-MAPK pathway epigenetic activation in cancer: miRNAs in action. Oncotarget.

[9] Yang S.-H., Sharrocks A.D., Whitmarsh A.J. (2013). MAP kinase signalling cascades and transcriptional regulation. Gene.

[10] Bray F., Ferlay J., Soerjomataram I., Siegel R.L., Torre L.A., Jemal A. (2018). Global cancer statistics 2018: GLOBOCAN estimates of incidence and mortality worldwide for 36 cancers in 185 countries. CA A Cancer J. Clin..

[11] Riihimäki M., Hemminki A., Sundquist K., Sundquist J., Hemminki K. (2016). Metastatic spread in patients with gastric cancer. Oncotarget.

[12] Kim H.S., Kim M.H., Jeong M., Hwang Y.S., Lim S.H., Shin B.A., Ahn B.W., Jung Y.D. (2004). EGCG blocks tumor promoter-induced MMP-9 expression via suppression of MAPK and AP-1 activation in human gastric AGS cells. Anticancer Res..

[13] Biagioni A., Skalamera I., Peri S., Schiavone N., Cianchi F., Giommoni E., Magnelli L., Papucci L. (2019). Update on gastric cancer treatments and gene therapies. Cancer Metastasis Rev..

[14] Hou F., Shi D.-B., Chen Y.-Q., Gao P. (2019). Human Epidermal Growth Factor Receptor-2 Promotes Invasion and Metastasis in Gastric Cancer by Activating Mitogen-activated Protein Kinase Signaling. Appl. Immunohistochem. Mol. Morphol..

[15] Laurén P. (1965). The two histological main types of gastric carcinoma: Diffuse and so-called intestinal-type carcinoma: An Attempt at a Histo-Clinical Classification. Acta Pathol. Microbiol. Scand..

[16] Tan P., Yeoh K.-G. (2015). Genetics and Molecular Pathogenesis of Gastric Adenocarcinoma. Gastroenterology.

[17] Hatakeyama M. (2014). Helicobacter pylori CagA and Gastric Cancer: A Paradigm for Hit-and-Run Carcinogenesis. Cell Host Microbe.

[18] Russi S., Verma H.K., Laurino S., Mazzone P., Storto G., Nardelli A., Zoppoli P., Calice G., La Rocca F., Sgambato A. (2019). Adapting and Surviving: Intra and Extra-Cellular Remodeling in Drug-Resistant Gastric Cancer Cells. Int. J. Mol. Sci..

[19] Dhillon A.S., Hagan S., Rath O., Kolch W. (2007). MAP kinase signalling pathways in cancer. Oncogene.

[20] Akter H., Park M., Kwon O.-S., Song E.J., Park W.-S., Kang M.-J. (2015). Activation of matrix metalloproteinase-9 (MMP-9) by neurotensin promotes cell invasion and migration through ERK pathway in gastric cancer. Tumour Biol..

[21] Baba K., Kitajima Y., Miyake S., Nakamura J., Wakiyama K., Sato H., Okuyama K., Kitagawa H., Tanaka T., Hiraki M. (2017). Hypoxia-induced ANGPTL4 sustains tumour growth and anoikis resistance through different mechanisms in scirrhous gastric cancer cell lines. Sci. Rep..

[22] Yang M., Huang C.-Z. (2015). Mitogen-activated protein kinase signaling pathway and invasion and metastasis of gastric cancer. World J. Gastroenterol..

[23] Han H., Lim J.W., Kim H. (2019). Lycopene Inhibits Activation of Epidermal Growth Factor Receptor and Expression of Cyclooxygenase-2 in Gastric Cancer Cells. Nutrients.

[24] Li W., Fan M., Chen Y., Zhao Q., Song C., Yan Y., Jin Y., Huang Z., Lin C., Wu J. (2015). Melatonin Induces Cell Apoptosis in AGS Cells Through the Activation of JNK and P38 MAPK and the Suppression of Nuclear Factor-Kappa B: A Novel Therapeutic Implication for Gastric Cancer. Cell. Physiol. Biochem..

[25] Liu Y., Fan D. (2019). Ginsenoside Rg5 induces G2/M phase arrest, apoptosis and autophagy via regulating ROS-mediated MAPK pathways against human gastric cancer. Biochem. Pharmacol..

[26] Wu L., Chen J., Qin Y., Mo X., Huang M., Ru H., Yang Y., Liu J., Lin Y. (2016). SATB2 suppresses gastric cancer cell proliferation and migration. Tumour Biol..

[27] Stecca B., Rovida E. (2019). Impact of ERK5 on the Hallmarks of Cancer. Int. J. Mol. Sci..

[28] Deng N., Goh L.K., Wang H., Das K., Tao J., Tan I.B., Zhang S., Lee M., Wu J., Lim K.H. (2012). A comprehensive survey of genomic alterations in gastric cancer reveals systematic patterns of molecular exclusivity and co-occurrence among distinct therapeutic targets. Gut.

[29] Pandian J., Panneerpandian P., Devanandan H.J., Sekar B.T., Balakrishnan K., Selvarasu K., Muthupandi K., Ganesan K. (2020). Identification of the targeted therapeutic potential of doxycycline for a subset of gastric cancer patients. Ann. N. Y. Acad. Sci..

[30] Kim H.J., Lee S., Chun K.H., Jeon J.Y., Han S.J., Kim D.J., Kim Y.S., Woo J.-T., Nam M.-S., Baik S.H. (2018). Metformin reduces the risk of cancer in patients with type 2 diabetes: An analysis based on the Korean National Diabetes Program Cohort. Medicine (Baltimore).

[31] Valaee S., Yaghoobi M.M., Shamsara M. (2017). Metformin inhibits gastric cancer cells metastatic traits through suppression of epithelial-mesenchymal transition in a glucose-independent manner. PLoS ONE.

[32] Lu C.-C., Chiang J.-H., Tsai F.-J., Hsu Y.-M., Juan Y.-N., Yang J.-S., Chiu H.-Y. (2019). Metformin triggers the intrinsic apoptotic response in human AGS gastric adenocarcinoma cells by activating AMPK and suppressing mTOR/AKT signaling. Int. J. Oncol..

[33] Li H., Chen C. (2018). Quercetin Has Antimetastatic Effects on Gastric Cancer Cells via the Interruption of uPA/uPAR Function by Modulating NF-κb, PKC-δ, ERK1/2, and AMPKα. Integr. Cancer Ther..

[34] Yan W., Chen S., Zhao Y., Ye X. (2018). Fisetin inhibits the proliferation of gastric cancer cells and induces apoptosis through suppression of ERK 1/2 activation. Oncol. Lett..

[35] Liang L., Amin A., Cheung W.-Y., Xu R., Yu R., Tang J., Yao X., Liang C. (2019). Parameritannin A-2 from Urceola huaitingii enhances doxorubicin-induced mitochondria-dependent apoptosis by inhibiting the PI3K/Akt, ERK1/2 and p38 pathways in gastric cancer cells. Chem. Biol. Interact..

[36] Teng F., Xu Z., Chen J., Zheng G., Zheng G., Lv H., Wang Y., Wang L., Cheng X. (2018). DUSP1 induces apatinib resistance by activating the MAPK pathway in gastric cancer. Oncol. Rep..

[37] Kitzen J.J.E.M., De Jonge M.J.A., Lamers C.H.J., Eskens F.A.L.M., Van der Biessen D., Van Doorn L., Ter Steeg J., Brandely M., Puozzo C., Verweij J. (2009). Phase I dose-escalation study of F60008, a novel apoptosis inducer, in patients with advanced solid tumours. Eur. J. Cancer.

[38] Lu H., Feng Y., Hu Y., Guo Y., Liu Y., Mao Q., Xue W. (2020). Spondin 2 promotes the proliferation, migration and invasion of gastric cancer cells. J. Cell. Mol. Med..

[39] Chen P., Chen G., Wang C., Mao C. (2019). RAB13 as a novel prognosis marker promotes proliferation and chemotherapeutic resistance in gastric cancer. Biochem. Biophys. Res. Commun..

[40] Yusefi A.R., Bagheri Lankarani K., Bastani P., Radinmanesh M., Kavosi Z. (2018). Risk Factors for Gastric Cancer: A Systematic Review. Asian Pac. J. Cancer Prev..

[41] Plummer M., Franceschi S., Vignat J., Forman D., De Martel C. (2015). Global burden of gastric cancer attributable to *Helicobacter pylori*: *Helicobacter pylori* in gastric cancer. Int. J. Cancer.

[42] International Agency for Research on Cancer (1994). Schistosomes, liver flukes and Helicobacter pylori. IARC Working Group on the Evaluation of Carcinogenic Risks to Humans. Lyon, 7–14 June 1994. IARC Monogr. Eval. Carcinog. Risks Hum..

[43] Chang W.-L., Yeh Y.-C., Sheu B.-S. (2018). The impacts of H. pylori virulence factors on the development of gastroduodenal diseases. J. Biomed. Sci..

[44] Akbari M., Tabrizi R., Kardeh S., Lankarani K.B. (2019). Gastric cancer in patients with gastric atrophy and intestinal metaplasia: A systematic review and meta-analysis. PLoS ONE.

[45] Ding S.-Z., Smith M.F., Goldberg J.B. (2008). *Helicobacter pylori* and mitogen-activated protein kinases regulate the cell cycle, proliferation and apoptosis in gastric epithelial cells. J. Gastroenterol. Hepatol..

[46] Hatakeyama M. (2006). Helicobacter pylori CagA—A bacterial intruder conspiring gastric carcinogenesis. Int. J. Cancer.

[47] Park J.Y., Forman D., Waskito L.A., Yamaoka Y., Crabtree J.E. (2018). Epidemiology of Helicobacter pylori and CagA-Positive Infections and Global Variations in Gastric Cancer. Toxins.

[48] Krisch L.M., Posselt G., Hammerl P., Wessler S. (2016). CagA Phosphorylation in Helicobacter pylori-Infected B Cells Is Mediated by the Nonreceptor Tyrosine Kinases of the Src and Abl Families. Infect. Immun..

[49] Selbach M., Paul F.E., Brandt S., Guye P., Daumke O., Backert S., Dehio C., Mann M. (2009). Host Cell Interactome of Tyrosine-Phosphorylated Bacterial Proteins. Cell Host Microbe..

[50] Mimuro H., Suzuki T., Tanaka J., Asahi M., Haas R., Sasakawa C. (2002). Grb2 is a key mediator of helicobacter pylori CagA protein activities. Mol. Cell.

[51] Chiba T., Marusawa H., Seno H., Watanabe N. (2008). Mechanism for gastric cancer development by *Helicobacter pylori* infection. J. Gastroenterol. Hepatol..

[52] Eftang L.L., Esbensen Y., Tannæs T.M., Bukholm I.R., Bukholm G. (2012). Interleukin-8 is the single most up-regulated gene in whole genome profiling of H. pylori exposed gastric epithelial cells. BMC Microbiol..

[53] Zarogoulidis P., Katsikogianni F., Tsiouda T., Sakkas A., Katsikogiannis N., Zarogoulidis K. (2014). Interleukin-8 and Interleukin-17 for Cancer. Cancer Investig..

[54] Macrì A., Versaci A., Loddo S., Scuderi G., Travagliante M., Trimarchi G., Teti D., Famulari C. (2006). Serum levels of interleukin 1beta, interleukin 8 and tumour necrosis factor alpha as markers of gastric cancer. Biomarkers.

[55] Ritter B., Kilian P., Reboll M.R., Resch K., DiStefano J.K., Frank R., Beil W., Nourbakhsh M. (2011). Differential Effects of Multiplicity of Infection on Helicobacter pylori-Induced Signaling Pathways and Interleukin-8 Gene Transcription. J. Clin. Immunol..

[56] Aihara M., Tsuchimoto D., Takizawa H., Azuma A., Wakebe H., Ohmoto Y., Imagawa K., Kikuchi M., Mukaida N., Matsushima K. (1997). Mechanisms involved in Helicobacter pylori-induced interleukin-8 production by a gastric cancer cell line, MKN45. Infect. Immun..

[57] Seo J.H., Lim J.W., Kim H. (2013). Differential Role of ERK and p38 on NF-κB Activation in Helicobacter pylori-Infected Gastric Epithelial Cells. J. Cancer Prev..

[58] Pathak S.K., Tavares R., De Klerk N., Spetz A.-L., Jonsson A.-B. (2013). Helicobacter pylori protein JHP0290 binds to multiple cell types and induces macrophage apoptosis via tumor necrosis factor (TNF)-dependent and independent pathways. PLoS ONE.

[59] Tavares R., Pathak S.K. (2017). Helicobacter pylori Secreted Protein HP1286 Triggers Apoptosis in Macrophages via TNF-Independent and ERK MAPK-Dependent Pathways. Front. Cell. Infect. Microbiol..

[60] Liu N., Wu Q., Wang Y., Sui H., Liu X., Zhou N., Zhou L., Wang Y., Ye N., Fu X. (2014). Helicobacter pylori promotes VEGF expression via the p38 MAPK-mediated COX-2-PGE2 pathway in MKN45 cells. Mol. Med. Rep..

[61] Chae K.-S., Ryu B.-K., Lee M.-G., Byun D.-S., Chi S.-G. (2002). Expression and mutation analyses of MKK4, a candidate tumour suppressor gene encoded by chromosome 17p, in human gastric adenocarcinoma. Eur. J. Cancer.

[62] Cunningham S.C., Gallmeier E., Hucl T., Dezentje D.A., Abdelmohsen K., Gorospe M., Kern S.E. (2006). Theoretical proposal: Allele dosage of MAP2K4/MKK4 could rationalize frequent 17p loss in diverse human cancers. Cell Cycle.

[63] Robinson V.L., Shalhav O., Otto K., Kawai T., Gorospe M., Rinker-Schaeffer C.W. (2008). Mitogen-activated protein kinase kinase 4/c-Jun NH2-terminal kinase kinase 1 protein expression is subject to translational regulation in prostate cancer cell lines. Mol. Cancer Res..

[64] Wang L., Pan Y., Dai J.L. (2004). Evidence of MKK4 pro-oncogenic activity in breast and pancreatic tumors. Oncogene.

[65] Wang P.-N., Huang J., Duan Y.-H., Zhou J.-M., Huang P.-Z., Fan X.-J., Huang Y., Wang L., Liu H.-L., Wang J.-P. (2017). Downregulation of phosphorylated MKK4 is associated with a poor prognosis in colorectal cancer patients. Oncotarget.

[66] Liang T., Zhang X., Xue W., Zhao S., Zhang X., Pei J. (2014). Curcumin induced human gastric cancer BGC-823 cells apoptosis by ROS-mediated ASK1-MKK4-JNK stress signaling pathway. Int. J. Mol. Sci..

[67] Hyland P.L., Lin S.-W., Hu N., Zhang H., Wang L., Su H., Wang C., Ding T., Tang Z.-Z., Fan J.-H. (2014). Genetic variants in fas signaling pathway genes and risk of gastric cancer. Int. J. Cancer.

[68] Wang G.G., Allis C.D., Chi P. (2007). Chromatin remodeling and cancer, Part I: Covalent histone modifications. Trends Mol. Med..

[69] Weichert W., Röske A., Gekeler V., Beckers T., Ebert M.P.A., Pross M., Dietel M., Denkert C., Röcken C. (2008). Association of patterns of class I histone deacetylase expression with patient prognosis in gastric cancer: A retrospective analysis. Lancet Oncol..

[70] Dong C., Sun J., Ma S., Zhang G. (2019). K-ras-ERK1/2 down-regulates H2A.XY142ph through WSTF to promote the progress of gastric cancer. BMC Cancer.

[71] Xu J., Tian F., Chen X., Liu Z., Wu C., Zhao Z. (2020). Ras-ERK1/2 signaling participates in the progression of gastric cancer through repressing Aurora B-mediated H1.4 phosphorylation at Ser27. J. Cell. Physiol..

[72] Huang Y., Shen X.J., Zou Q., Wang S.P., Tang S.M., Zhang G.Z. (2011). Biological functions of microRNAs: A review. J. Physiol. Biochem..

[73] O’Brien J., Hayder H., Zayed Y., Peng C. (2018). Overview of MicroRNA Biogenesis, Mechanisms of Actions, and Circulation. Front. Endocrinol..

[74] Pan H.-W., Li S.-C., Tsai K.-W. (2013). MicroRNA dysregulation in gastric cancer. Curr. Pharm. Des..

[75] Alessandrini L., Manchi M., De Re V., Dolcetti R., Canzonieri V. (2018). Proposed Molecular and miRNA Classification of Gastric Cancer. Int. J. Mol. Sci..

[76] Zhang C.-Z., Han L., Zhang A.-L., Fu Y.-C., Yue X., Wang G.-X., Jia Z.-F., Pu P.-Y., Zhang Q.-Y., Kang C.-S. (2010). MicroRNA-221 and microRNA-222 regulate gastric carcinoma cell proliferation and radioresistance by targeting PTEN. BMC Cancer.

[77] Zhang B.G., Li J.F., Yu B.Q., Zhu Z.G., Liu B.Y., Yan M. (2012). microRNA-21 promotes tumor proliferation and invasion in gastric cancer by targeting PTEN. Oncol. Rep..

[78] Tsukamoto Y., Nakada C., Noguchi T., Tanigawa M., Nguyen L.T., Uchida T., Hijiya N., Matsuura K., Fujioka T., Seto M. (2010). MicroRNA-375 is downregulated in gastric carcinomas and regulates cell survival by targeting PDK1 and 14-3-3zeta. Cancer Res..

[79] Ding L., Xu Y., Zhang W., Deng Y., Si M., Du Y., Yao H., Liu X., Ke Y., Si J. (2010). MiR-375 frequently downregulated in gastric cancer inhibits cell proliferation by targeting JAK2. Cell Res..

[80] Takagi T., Iio A., Nakagawa Y., Naoe T., Tanigawa N., Akao Y. (2009). Decreased Expression of MicroRNA-143 and -145 in Human Gastric Cancers. Oncology.

[81] Zhou J., Chen Q. (2019). Poor expression of microRNA-135b results in the inhibition of cisplatin resistance and proliferation and induces the apoptosis of gastric cancer cells through MST1-mediated MAPK signaling pathway. FASEB J..

[82] Lang N., Liu M., Tang Q.-L., Chen X., Liu Z., Bi F. (2010). Effects of microRNA-29 family members on proliferation and invasion of gastric cancer cell lines. Chin J. Cancer.

[83] Hashimoto Y., Akiyama Y., Otsubo T., Shimada S., Yuasa Y. (2010). Involvement of epigenetically silenced microRNA-181c in gastric carcinogenesis. Carcinogenesis.

[84] Di Gesualdo F., Capaccioli S., Lulli M. (2014). A pathophysiological view of the long non-coding RNA world. Oncotarget.

[85] St Laurent G., Shtokalo D., Dong B., Tackett M.R., Fan X., Lazorthes S., Nicolas E., Sang N., Triche T.J., McCaffrey T.A. (2013). VlincRNAs controlled by retroviral elements are a hallmark of pluripotency and cancer. Genome Biol..

[86] Kung J.T.Y., Colognori D., Lee J.T. (2013). Long Noncoding RNAs: Past, Present, and Future. Genetics.

[87] Yang F., Bi J., Xue X., Zheng L., Zhi K., Hua J., Fang G. (2012). Up-regulated long non-coding RNA H19 contributes to proliferation of gastric cancer cells. FEBS J..

[88] Yan J., Zhang Y., She Q., Li X., Peng L., Wang X., Liu S., Shen X., Zhang W., Dong Y. (2017). Long Noncoding RNA H19/miR-675 Axis Promotes Gastric Cancer via FADD/Caspase 8/Caspase 3 Signaling Pathway. Cell. Physiol. Biochem..

[89] Yang W., Redpath R., Zhang C., Ning N. (2018). Long non-coding RNA H19 promotes the migration and invasion of colon cancer cells via MAPK signaling pathway. Oncol. Lett..

[90] Du T., Zhang B., Zhang S., Jiang X., Zheng P., Li J., Yan M., Zhu Z., Liu B. (2016). Decreased expression of long non-coding RNA WT1-AS promotes cell proliferation and invasion in gastric cancer. Biochim. Biophys. Acta (BBA) Mol. Basis Dis..

[91] Dallosso A.R., Hancock A.L., Malik S., Salpekar A., King-Underwood L., Pritchard-Jones K., Peters J., Moorwood K., Ward A., Malik K.T.A. (2007). Alternately spliced WT1 antisense transcripts interact with WT1 sense RNA and show epigenetic and splicing defects in cancer. RNA.

[92] Sun D., Li X., He Y., Li W., Wang Y., Wang H., Jiang S., Xin Y. (2016). YAP1 enhances cell proliferation, migration, and invasion of gastric cancer in vitro and in vivo. Oncotarget.

[93] Yu F., Chen B., Dong P., Zheng J. (2017). HOTAIR Epigenetically Modulates PTEN Expression via MicroRNA-29b: A Novel Mechanism in Regulation of Liver Fibrosis. Mol. Ther..

[94] Xun J., Wang C., Yao J., Gao B., Zhang L. (2019). Long Non-Coding RNA HOTAIR Modulates KLF12 to Regulate Gastric Cancer Progression via PI3K/ATK Signaling Pathway by Sponging miR-618. OTT.

[95] Liu S., Yan G., Zhang J., Yu L. (2018). Knockdown of Long Noncoding RNA (lncRNA) Metastasis-Associated Lung Adenocarcinoma Transcript 1 (MALAT1) Inhibits Proliferation, Migration, and Invasion and Promotes Apoptosis by Targeting miR-124 in Retinoblastoma. Oncol. Res..

[96] Li Y., Li D., Zhao M., Huang S., Zhang Q., Lin H., Wang W., Li K., Li Z., Huang W. (2018). Long noncoding RNA SNHG6 regulates p21 expression via activation of the JNK pathway and regulation of EZH2 in gastric cancer cells. Life Sci..

[97] Yan K., Tian J., Shi W., Xia H., Zhu Y. (2017). LncRNA SNHG6 is Associated with Poor Prognosis of Gastric Cancer and Promotes Cell Proliferation and EMT through Epigenetically Silencing p27 and Sponging miR-101-3p. Cell. Physiol. Biochem..

[98] Pulati N., Zhang Z., Gulimilamu A., Qi X., Yang J. (2019). HPV16 ^+^ -miRNAs in cervical cancer and the anti-tumor role played by miR-5701. J. Gene Med..

[99] Cui L., Nai M., Zhang K., Li L., Li R. (2019). lncRNA WT1-AS inhibits the aggressiveness of cervical cancer cell via regulating p53 expression via sponging miR-330-5p. CMAR.

[100] Qu C.-X., Shi X.-C., Bi H., Zhai L.-Q., Yang Q. (2019). LncRNA AOC4P affects biological behavior of gastric cancer cells through MAPK signaling pathway. Eur. Rev. Med. Pharmacol. Sci..

[101] Zhang H., Zhang Z., Wang D. (2019). Epigenetic regulation of IncRNA KCNKI5-ASI in gastric cancer. CMAR.

[102] Li P., Xue W.-J., Feng Y., Mao Q.-S. (2016). Long non-coding RNA CASC2 suppresses the proliferation of gastric cancer cells by regulating the MAPK signaling pathway. Am. J. Transl. Res..

[103] Yang H., Liu Z., Yuan C., Zhao Y., Wang L., Hu J., Xie D., Wang L., Chen D. (2015). Elevated JMJD1A is a novel predictor for prognosis and a potential therapeutic target for gastric cancer. Int. J. Clin. Exp. Pathol..

[104] Zhang Y., Ma M., Liu W., Ding W., Yu H. (2014). Enhanced expression of long noncoding RNA CARLo-5 is associated with the development of gastric cancer. Int. J. Clin. Exp. Pathol..

[105] Dou G., Zhang J., Wang P., Wang J., Sun G. (2019). Long Intergenic Non-Protein-Coding RNA 01138 Accelerates Tumor Growth and Invasion in Gastric Cancer by Regulating miR-1273e. Med. Sci. Monit..

[106] Zhang H., Cai Y., Zheng L., Zhang Z., Lin X., Jiang N. (2018). Long noncoding RNA NEAT1 regulate papillary thyroid cancer progression by modulating miR-129-5p/ *KLK7* expression. J. Cell Physiol..

[107] Fu R., Wang X., Hu Y., Du H., Dong B., Ao S., Zhang L., Sun Z., Zhang L., Lv G. (2019). Solamargine inhibits gastric cancer progression by regulating the expression of lncNEAT1_2 via the MAPK signaling pathway. Int. J. Oncol..

[108] Wang X., Liang Q., Zhang L., Gou H., Li Z., Chen H., Dong Y., Ji J., Yu J. (2019). C8orf76 Promotes Gastric Tumorigenicity and Metastasis by Directly Inducing lncRNA DUSP5P1 and Associates with Patient Outcomes. Clin. Cancer Res..

[109] Li J.-F., Li W.-H., Xue L.-L., Zhang Y. (2019). Long non-coding RNA PICART1 inhibits cell proliferation by regulating the PI3K/AKT and MAPK/ERK signaling pathways in gastric cancer. Eur. Rev. Med. Pharmacol. Sci..

[110] Wu L., Yin J.-H., Guan Y.-Y., Liu H.-L., Shen H.-L., Wang X.-J., Han B.-H., Zhou M.-W., Gu X.-D. (2018). A long noncoding RNA MAP3K1-2 promotes proliferation and invasion in gastric cancer. OTT.

[111] Li D., Yang M., Liao A., Zeng B., Liu D., Yao Y., Hu G., Chen X., Feng Z., Du Y. (2018). Linc00483 as ceRNA regulates proliferation and apoptosis through activating MAPKs in gastric cancer. J. Cell Mol. Med..

[112] Luo Y., Wang C., Yong P., Ye P., Liu Z., Fu Z., Lu F., Xiang W., Tan W., Xiao J. (2017). Decreased expression of the long non-coding RNA SLC7A11-AS1 predicts poor prognosis and promotes tumor growth in gastric cancer. Oncotarget.

